# Runx2 controls the osteogenic fate of growth plate chondrocytes

**DOI:** 10.1016/j.gendis.2024.101453

**Published:** 2024-11-09

**Authors:** Daofu Zeng, Jiamin Yu, Ke Lu, Dan Yi, Zhidao Xia, Ling Qin, Guozhi Xiao, Xiao Yang, Liping Tong, Di Chen

**Affiliations:** Research Center for Computer-aided Drug Discovery, Shenzhen Institute of Advanced Technology, Chinese Academy of Sciences, Shenzhen, Guangdong 518055, China; Faculty of Pharmaceutical Sciences, Shenzhen University of Advanced Technology, Shenzhen, Guangdong 518055, China; Research Center for Computer-aided Drug Discovery, Shenzhen Institute of Advanced Technology, Chinese Academy of Sciences, Shenzhen, Guangdong 518055, China; Faculty of Pharmaceutical Sciences, Shenzhen University of Advanced Technology, Shenzhen, Guangdong 518055, China; University of Chinese Academy of Sciences, Chinese Academy of Sciences, Beijing 100049, China; Research Center for Computer-aided Drug Discovery, Shenzhen Institute of Advanced Technology, Chinese Academy of Sciences, Shenzhen, Guangdong 518055, China; Faculty of Pharmaceutical Sciences, Shenzhen University of Advanced Technology, Shenzhen, Guangdong 518055, China; Institute of Life Science, Swansea University Medical School, Swansea, SA2 8PP, UK; Musculoskeletal Research Laboratory of Department of Orthopaedics & Traumatology and Innovative Orthopaedic Biomaterial & Drug Translational Research Laboratory, Li Ka Shing Institute of Health Sciences, The Chinese University of Hong Kong, Hong Kong 518172, China; School of Medicine, Southern University of Science and Technology, Shenzhen, Guangdong 518055, China; State Key Laboratory of Proteomics, Genetic Laboratory of Development and Diseases, Institute of Biotechnology, Beijing 100071, China; Research Center for Computer-aided Drug Discovery, Shenzhen Institute of Advanced Technology, Chinese Academy of Sciences, Shenzhen, Guangdong 518055, China; Research Center for Computer-aided Drug Discovery, Shenzhen Institute of Advanced Technology, Chinese Academy of Sciences, Shenzhen, Guangdong 518055, China; Faculty of Pharmaceutical Sciences, Shenzhen University of Advanced Technology, Shenzhen, Guangdong 518055, China

The origin of bone marrow osteoblasts is not totally understood. Recent findings demonstrated that bone marrow osteoblasts could be derived from a subpopulation of hypertrophic Col2^+^/Col10^+^ chondrocytes which migrate from the growth plate into the bone marrow cavity underneath the growth plate and dedifferentiate into mesenchymal progenitor cells and then differentiate into mature osteoblasts.^1^ This process is called chondrocyte-osteoblast transdifferentiation. This type of osteoblast participates in bone formation and is involved in maintaining bone remodeling, especially in the epiphyseal and diaphyseal regions of long bone. Several growth factors, such as Ihh, PTH, and Wnt signaling molecules have been demonstrated to play a critical role in the regulation of chondrocyte-osteoblast transdifferentiation^2^; however, the role of Runx2, the key transcription factor controlling skeletal development,^3^ in chondrocyte-osteoblast transdifferentiation has not been fully defined.

To examine the role of Runx2 in chondrocyte transdifferentiation in *Col2*-expressing cells, we first confirm that *Col2*-expressing cells indeed can migrate into the bone marrow cavity. So, we generated *ZsGreen-tdTomato*^Col2CreER^ mice, and the expression of the reporter gene is controlled by the *Col2* promoter. We administered tamoxifen (0.75 mg/10 g body weight) to these mice at the postnatal P7 stage by intraperitoneal injections for three consecutive days. The *Col2*-labeling cells showed migration to the bone marrow cavity in a time-dependent manner (Fig. S1), indicating that *Col2*-expressing cells could migrate into the bone marrow cavity. We then determined the role of Runx2 in chondrocyte transdifferentiation by generating *Runx2*^Col2CreER^ conditional knockout (*Runx2* cKO) mice through breeding *Runx2*^flox/flox^ mice^4^ with *Col2-CreER* transgenic mice.^5^ Deletion of *Runx2* in *Col2*-expressing cells in growth plate cartilage causes animal growth delay (Fig. S2) and defects in growth plate chondrocyte differentiation. The hypertrophic zone of growth plate cartilage was significantly expanded (Fig. 1A–C). This could be due to the acceleration of chondrocyte hypertrophy or the delay of chondrocyte transdifferentiation after the loss of *Runx2* in *Col2*-expressing cells. The numbers of Col-X-positive cells were significantly increased (Fig. S3), suggesting that the process of chondrocyte hypertrophy was increased.

**Figure 1:**
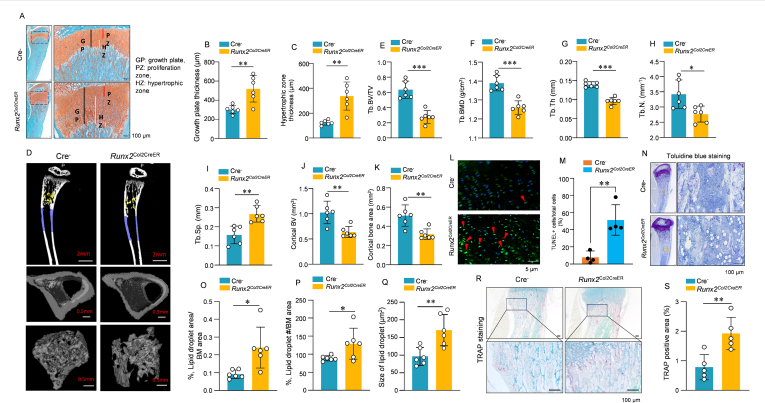
Runx2 controls the osteogenic fate of growth plate chondrocytes. **(A–C)** Growth plate cartilage expansion in *Runx2*^Col2CreER^ (*Runx2* cKO) mice. Histological analysis showed growth plate cartilage defects in *Runx2* cKO mice, including significantly enlarged growth cartilage thickness and growth plate hypertrophic zone. **(D**–**K)** Trabecular bone loss in *Runx2* cKO mice. Micro-CT analysis showed that bone volume (BV), bone mineral density (BMD), and trabecular thickness (Tb.Th.) were significantly decreased (D–H). In contrast, trabecular separation (Tb.Sp.) was significantly increased (I). In addition, cortical bone volume (BV) and cortical bone area (BA) were significantly decreased **(J, K)**. **(L, M)** Terminal deoxynucleotidyl transferase dUTP nick-end labeling (TUNEL) assay demonstrated that apoptosis cell numbers were significantly increased in *Runx2* cKO mice. **(N–Q)** Toluidine blue staining showed that adipocyte numbers, lipid droplet numbers, and average lipid droplet area were significantly increased in *Runx2* cKO mice. **(R, S)** TRAP staining showed that TRAP-positive cell numbers were increased in *Runx2* cKO mice.

In addition to the defects in growth plate cartilage development, *Runx2* cKO mice also showed trabecular bone loss in the bone marrow cavity (Fig. 1D). Trabecular bone volume, bone mineral density, trabecular thickness, and trabecular numbers were significantly reduced (Fig. 1E–H). In contrast, trabecular separation was significantly increased (Fig. 1I). In addition, cortical bone volume and cortical bone area were also significantly decreased in *Runx2* cKO mice (Fig. 1J, K). We also determined changes in cell apoptosis in *Runx2* cKO mice by performing terminal deoxynucleotidyl transferase dUTP nick-end labeling (TUNEL) assays. We found that apoptotic cell numbers were significantly increased, especially in the hypertrophic zone of *Runx2* cKO mice (Fig. 1M). Consistent with the bone loss phenotype, expression of osteoblast marker genes was significantly decreased in 4- and 6-week-old *Runx2* cKO mice (Fig. S4, 5). Since transdifferentiated progenitor cells could also differentiate into adipocytes,^2^ we also determined adipocyte formation in *Runx2* cKO mice and found that adipocyte formation was significantly increased in *Runx2* cKO mice (Fig. 1N–Q). We determined the expression of adipocyte marker genes and found that expression of lipoprotein lipase (*LPL*) and CCAAT enhancer binding protein β (*C-EBP-β*) was significantly up-regulated; however, expression of perilipin 1 (*PLIN1*) and peroxisome proliferator activated receptor gamma (*PPAR-γ*) was not significantly changed in bone marrow stromal cells of 4- and 6-week-old *Runx2* cKO mice (Fig. S6, 7). We further determined changes in osteoclast formation by performing tartrate-resistant acid phosphatase (TRAP) staining and found that the numbers of TRAP-positive osteoclasts were significantly increased in *Runx2* cKO mice (Fig. 1R, S). We also examined changes in osteoclast marker genes in bone marrow stromal cells, such as *Rankl* and *Opg*, and found no significant changes in these genes in bone marrow stromal cells of *Runx2* cKO mice (Fig. S8). Our findings demonstrated that Runx2 plays a key role in controlling chondrocyte-osteoblast transdifferentiation. It has been demonstrated that Ihh, PTH, and Wnt signaling pathways regulate chondrocyte-osteoblast transdifferentiation.^2^ However, it is not clear if Runx2 is involved in these signaling pathways, although our findings demonstrated that Runx2 plays a key role in controlling chondrocyte-osteoblast transdifferentiation. The role of Runx2 in Ihh, PTH, and Wnt signaling during chondrocyte-osteoblast transdifferentiation requires further investigation.

Our studies using *Col2-CreER* mice and findings from other laboratories using *Col10-CreER* mice^1^ clearly indicate that only a subpopulation of hypertrophic chondrocytes is involved in chondrocyte-osteoblast transdifferentiation. However, it is not known how the functions of progenitor cells from the periosteum and the hypertrophic chondrocytes in the growth plate are coordinated during bone remodeling, which also requires in-depth investigation.

## Conflict of interests

The authors declared no competing interests.

## Funding

This project was supported by the 10.13039/501100012166National Key Research and Development Program of China (No. 2022YFA1207500), the 10.13039/501100001809National Natural Science Foundation of China (No. 82394445 to D.C. and L.T.; 82250710174 and 82030067 to D.C.; 82161160342 to D.C. and L.Q.), and the Shenzhen Science and Technology Research Funding (No. JCYJ20220818101414032 to L.T.; JSGGKQTD20210831174330015 to D.C.).

## Author contributions

**Daofu Zeng:** Conceptualization, Data curation, Formal analysis, Investigation. **Jiamin Yu:** Data curation, Investigation, Methodology, Project administration. **Ke Lu:** Conceptualization, Software. **Dan Yi:** Formal analysis, Methodology. **Zhidao Xia:** Supervision, Validation. **Ling Qin:** Supervision, Validation, Visualization. **Guozhi Xiao:** Supervision, Validation, Visualization. **Xiao Yang:** Supervision, Validation, Visualization. **Liping Tong:** Supervision, Validation, Visualization. **Di Chen:** Supervision, Validation, Visualization, Writing – original draft, Writing – review & editing.
