# Supplementary material for: Runx2 controls the osteogenic fate of growth plate chondrocytes
Source: Genes Dis. 2024 Nov 9;12(3):101453. doi: 10.1016/j.gendis.2024.101453 (PMC11761896; doi:10.1016/j.gendis.2024.101453)
Supplement: Multimedia component 1 [file mmc1.docx]

This file includes:

Supplementary Materials and Methods

Supplementary Figures S1 to S8

**Materials and methods**

Animal study

*Col2-CreER* mice were previously generated in our lab and *Runx2^flox/flox^* mice and *ZsGreen-tdTomato* reporter mice were purchased from GemPharmatech Co., Ltd. To obtain *Runx2^Col2CreER^* mice, *Runx2^flox/flox^* mice were crossed with *Col2-CreER* mice. To obtain *ZsGreen-tdTomato^Col2-CreER^* mice, *ZsGreen-tdTomato* reporter mice were crossed with *Col2-CreER* mice. All mice were placed in specific pathogen-free (SPF) ventilated cages and maintained at 22°C, 12 h alternating light and dark, and stable humidity (50% ± 10%) conditions. All mice had free access to food and water. DNA extracted from the tails of newborn mice and mice genotypes were detected by PCR. The primer sequences are shown in Table 1. To delete *Runx2* gene or to activate the *tdTomato* reporter gene, *Runx2^Col2CreER^* mice and *ZsGreen-tdTomato^Col2-CreER^* mice were treated with tamoxifen (Sigma; Louis, MO, USA) at 1 week of age (i.p. injection, 0.75 mg/10 g body weight for 5 days) and mice were sacrificed at 4 weeks of age. The samples of *ZsGreen-tdTomato^Col2-CreER^* mice were collected and analyzed 1, 5 and 10 days after tamoxifen induction. The experimental protocol of this study was approved by the Ethics Committee of Shenzhen Institute of Advanced Technology, Chinese Academy of Sciences (Project Ethics Number: SIAT-IACUC-20230403-YYS-JSYWZX-LK-A2190-01).

Micro-computed tomography (Micro-CT) and analysis

After the mice were euthanized, the bone tissue was fixed in 10% neutral formalin for 24 hours. Bone tissue was scanned using a NEMO Micro-CT scanner (Shanghai Pingsheng Health Medical Co., Ltd., Shanghai, China) with a source voltage of 90 kV, a current of 70 μA, and a resolution of 10 μm. Bone parameters were calculated for the bone tissue below the tibial growth plate and the fourth lumbar vertebra, including trabecular bone volume per unit tissue volume (BV/TV), trabecular bone density (BMD), trabecular thickness (Tb.Th.), trabecular number (Tb.N.) and trabecular separation (Tb.Sp.), as well as cortical bone area (Ct.Ar.), cortical bone volume (Ct. BV).

Histological analysis

For histological analysis, the mouse tibia tissue was fixed in 10% neutral formalin for 24 hours and decalcified with 14% EDTA for two weeks. The decalcified tissue was embedded in paraffin and then cut into 5 μm sections. After dewaxing and rehydration, tissue sections were stained with safranine o-fast green for histological analysis to evaluate growth plate development. Toluidine blue staining was used to analyze the number and size of fat vacuoles in the bone marrow cavity to evaluate adipogenesis in the bone marrow cavity.

Immunohistochemical (IHC) staining

For IHC staining, after rehydration and antigen retrieval, the tissue was sequentially treated with endogenous peroxidase blocking buffer (Beyotime, China), 0.5% Triton X-100, and blocking with 10% goat serum, then incubated with primary antibody (Collagen X polyclonal Antibody, Invitrogen, 1:100; Osteocalcin, Abcam, 1:100) overnight at 4°C. Then, the sections were incubated with secondary biotinylated antibodies (Goat Anti-Rabbit IgG (H+L), HRP, BE0101-100UL) for 1h, and after being treated with VECTASTAIN Elite ABC Kit, IHC signals were displayed with Imm PACT DAB Peroxidase Substrate. All stained sections were captured on a Leica microscope (DM2000 LED, Shanghai) using Leica Application Suite X software and analyzed using ImageJ software.

Tartrate-resistant acid phosphatase (TRAP) staining

For TRAP staining, paraffin tissue sections were stained with TRAP and methyl green following the instructions of the TRAP staining kit (Servicebio, G1050-50T) to determine changes in osteoclast numbers.

RNA extraction and real-time quantitative PCR

RNA was extracted from mouse tibia and femur bone marrow. Specifically, the tibia and femur were collected immediately after the mice were euthanized, and the soft tissues were removed. Rinse the tibia and femur in PBS, cut off the bone tissue at both ends, and then rinse out the bone marrow cells with PBS. Total RNA was extracted from washed bone marrow cells using Trizol reagent (Invitrogen, USA), and use RT reagent kit (HiScript III All-in-one RT SuperMix Perfect for qPCR, Vazyme). Reverse transcription was performed to obtain cDNA. Then qPCR amplification was performed using SYBR-Green according to the instructions. Different samples were normalized and relative fold changes were determined by the 2^-(ΔΔCt)^ method. The qPCR primer sequences used in the experiment are shown in Table 2.

Terminal deoxynucleotidyl transferase dUTP nick-end labeling (TUNEL) assay

Briefly, paraffin-embedded tissue sections (4 µm) underwent deparaffinization process in xylene, subsequently being rehydrated through a graded series of decreasing ethanol concentrations. Following this, the TUNEL staining protocol provided by the kit (Beyotime, C1086) was adhered to, enabling the observation of change in growth plate.

Statistical analysis

Statistical analysis was performed using Prism GraphPad Prim 8.0 software. Unpaired two-tailed Student's *t*-test was used to analyze differences between the two groups and a one-way analysis of variance (ANOVA) was used to analyze the differences of multiple groups. All data are expressed as mean ± S.D.

**Table 1**: Primer used for genotyping.

| Genes | forward primers | reverse primers |
| --- | --- | --- |
| *Runx2^flox/flox^* | AAGCCGGGGAAAGGAAGGACT | AGTCCCTGGCGGTGGCTGCAA |
| *Col2^CreER^* | ATCCGAAAAGAAAACGTTGA | ATCCAGGTTACGGATATAGT |
| *ZsGreen* | CCTCCTCTCCTGACTACTCCCAGTC | TCACAGAAACCATATGGCGCTCC |

**Table 2**: Primer used for qPCR.

| Genes | Forward primers | Reverse primers |
| --- | --- | --- |
| *β-actin* | CATGTACGTTGCTATCCAGGC | CTCCTTAATGTCACGCACGAT |
| *Ocn* | GAACAGACAAGTCCCACACAG | GAGCTGCTGTGACATCCATAC |
| *Osx* | ATGGCGTCCTCTCTGCTTG | TGAAAGGTCAGCGTATGGCTT |
| *Alp* | CAAGGATGCTGGGAAGTCCG | CGGATAACGAGATGCCACCA |
| *Runx2* | CCGTGGCCTTCAAGGTTGT | TTCATAACAGCGGAGGCATTT |
| *Opn* | CTGGCAGCTCAGAGGAGAAG | CAGCATTCTGTGGCGCAAG |
| *Col1α1* | GCTCCTCTTAGGGGCCACT | CCACGTCTCACCATTGGGG |
| *Rankl* | ACCAGCATCAAAATCCCAAG | TTTGAAAGCCCCAAAGTACG |
| *OPG* | TGACCTCTGTGAAAGCAGCGTG | GCCCTTCAAGGTGTCTTGGTCA |
| *LPL* | CCCTCTCTTACAAGCCCATCA | GAGCCAGTCTGGTAGTACATCA |
| *PLIN1* | TGTGCAATGCCTATGAGAAGG | AGGGCGGGGATCTTTTCCT |
| *PPAR-γ* | TTTTCAAGGGTGCC AGTTTC | AATCCTTGGCCCTCTGAGAT |
| *C-EBP-β* | GTTTCGGGACTTGATGCAAT | GCCCGGCTAGACAGTTACAC |


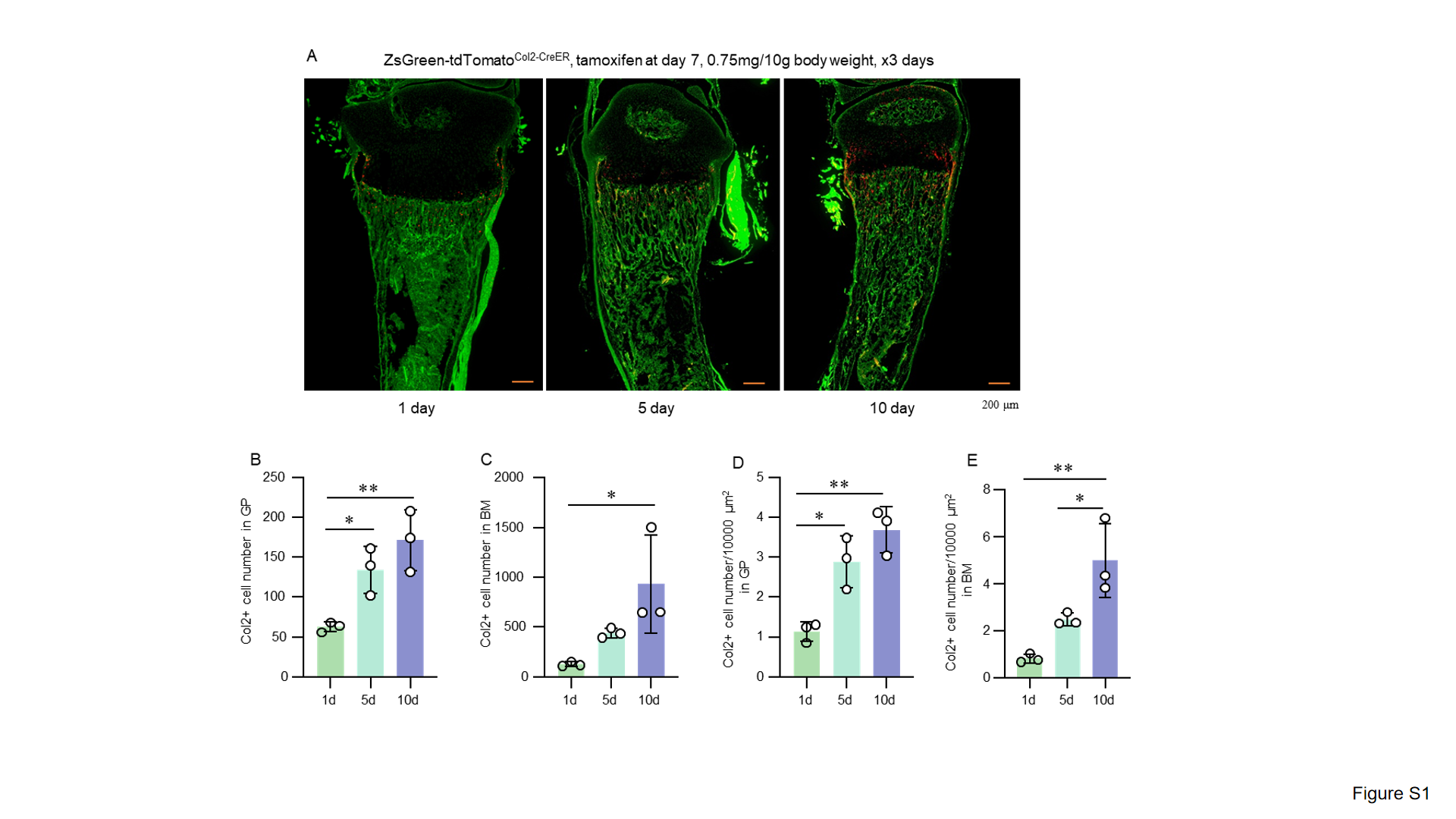


**Figure S1.** Migration of growth plate Col2+ chondrocytes into bone marrow stromal (BMS) cavity underneath the growth plate. *Col2-CreER* mice were bred with *ZsGreen-tdTomato* reporter mice to generate *ZsGreen-tdTomato^Col2-CreER^* mice. Tamoxifen was administered into *ZsGreen-tdTomato^Col2-CreER^* mice at the concentration of 0.75 mg/10g body weight, starting at postnatal P7 for consecutive 3 days and mice were sacrificed 1, 5 and 10 days after tamoxifen induction. With more Col2+ cells were labeled in growth plate (A-C), more Col2+ cells were migrated into bone marrow cavity (A, D, E).

**
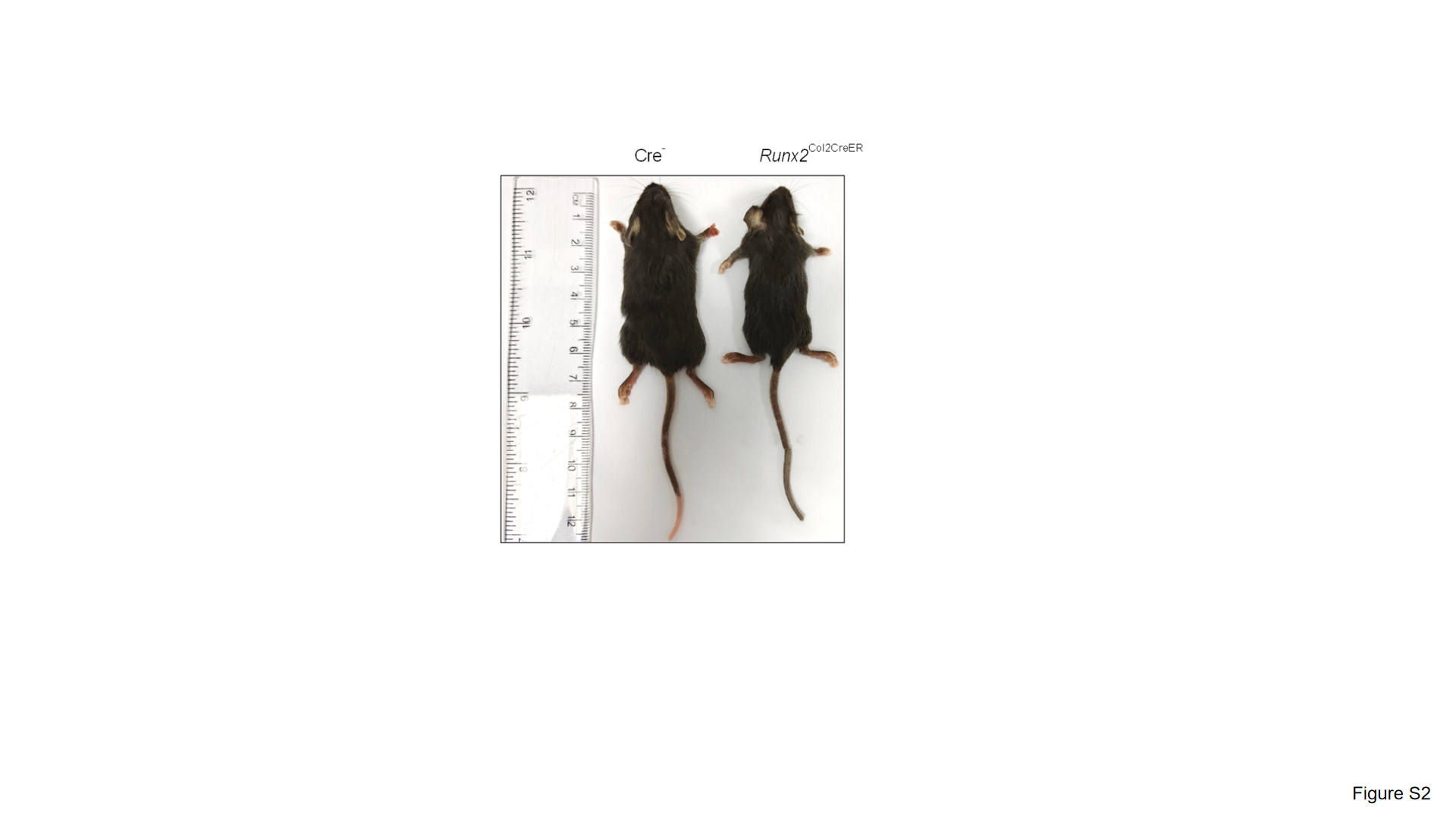
**

**Figure S2.** The growth retardation in *Runx2^Col2CreER^* conditional knockout (*Runx2* cKO) mice.


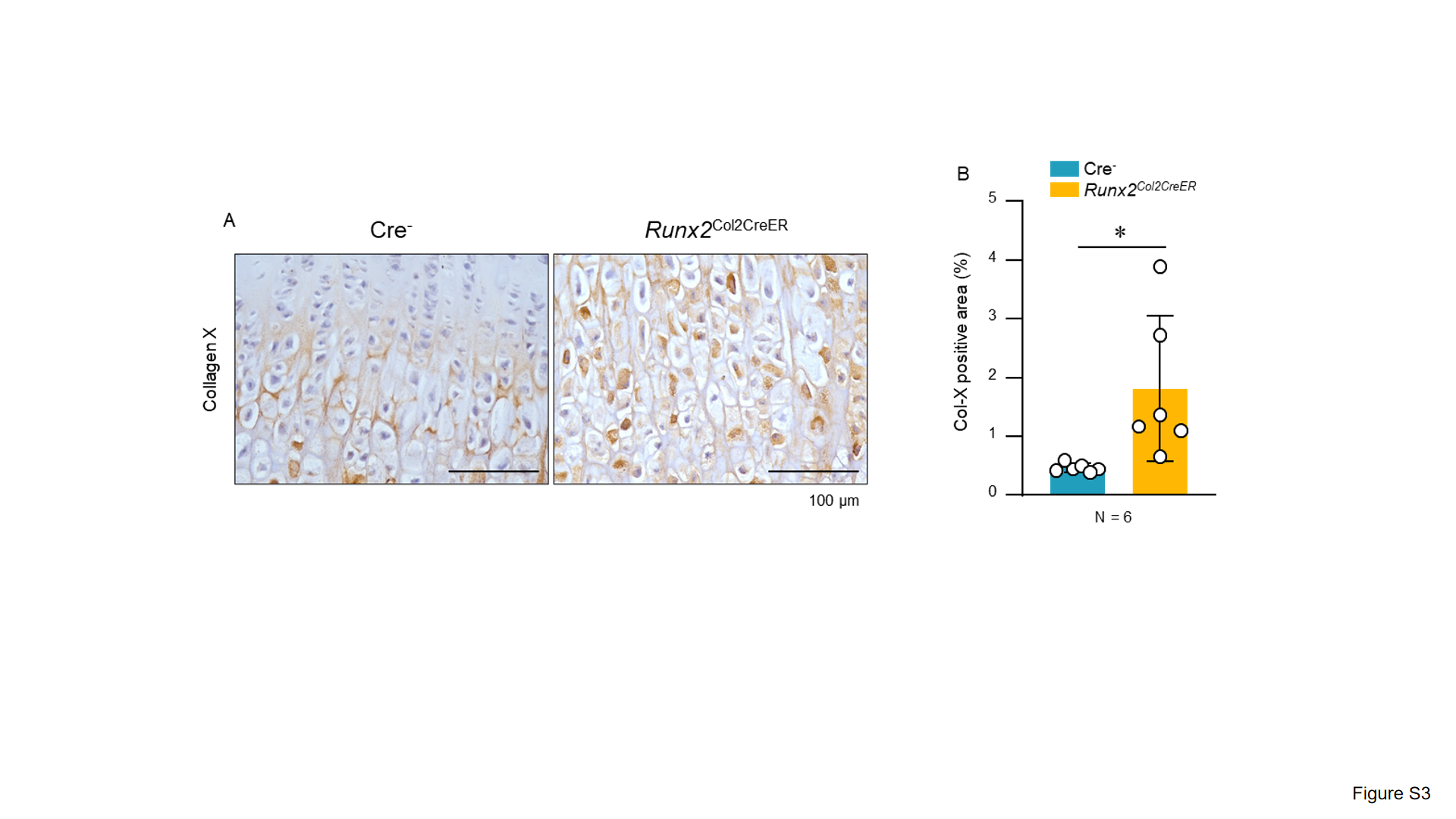


**Figure S3.** The size of growth plate cartilage and Col-X+ cell numbers were significantly increased in *Runx2^Col2CreER^* (*Runx2* cKO) mice.


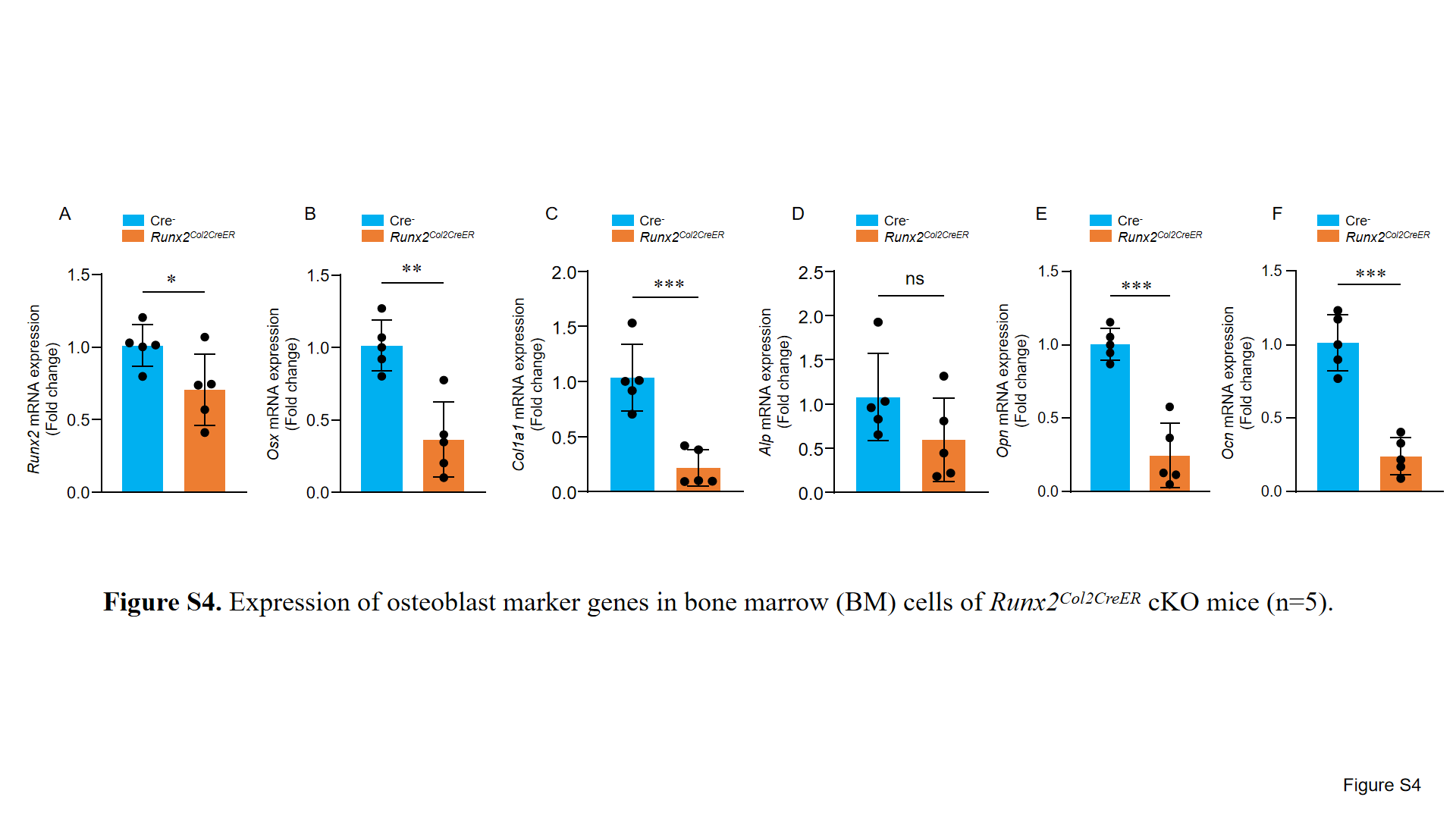


**Figure S4.** Expression of osteoblast marker genes in bone marrow stromal (BMS) cells. BMS cells were isolated from 4-week-old Cre^-^ and *Runx2^Col2CreER^* (*Runx2* cKO) mice. Expression of *Runx2*, *Osterix* (*Osx*), *Col1a1*, *Osteopontin* (*Opn*) and *Osteocalcin* (*Ocn*) was significantly reduced (A-C, E, F); while expression of *alkaline phosphatase* (*Alp*) was not significantly changed in BMS cells of *Runx2* cKO mice (D) (n=5).


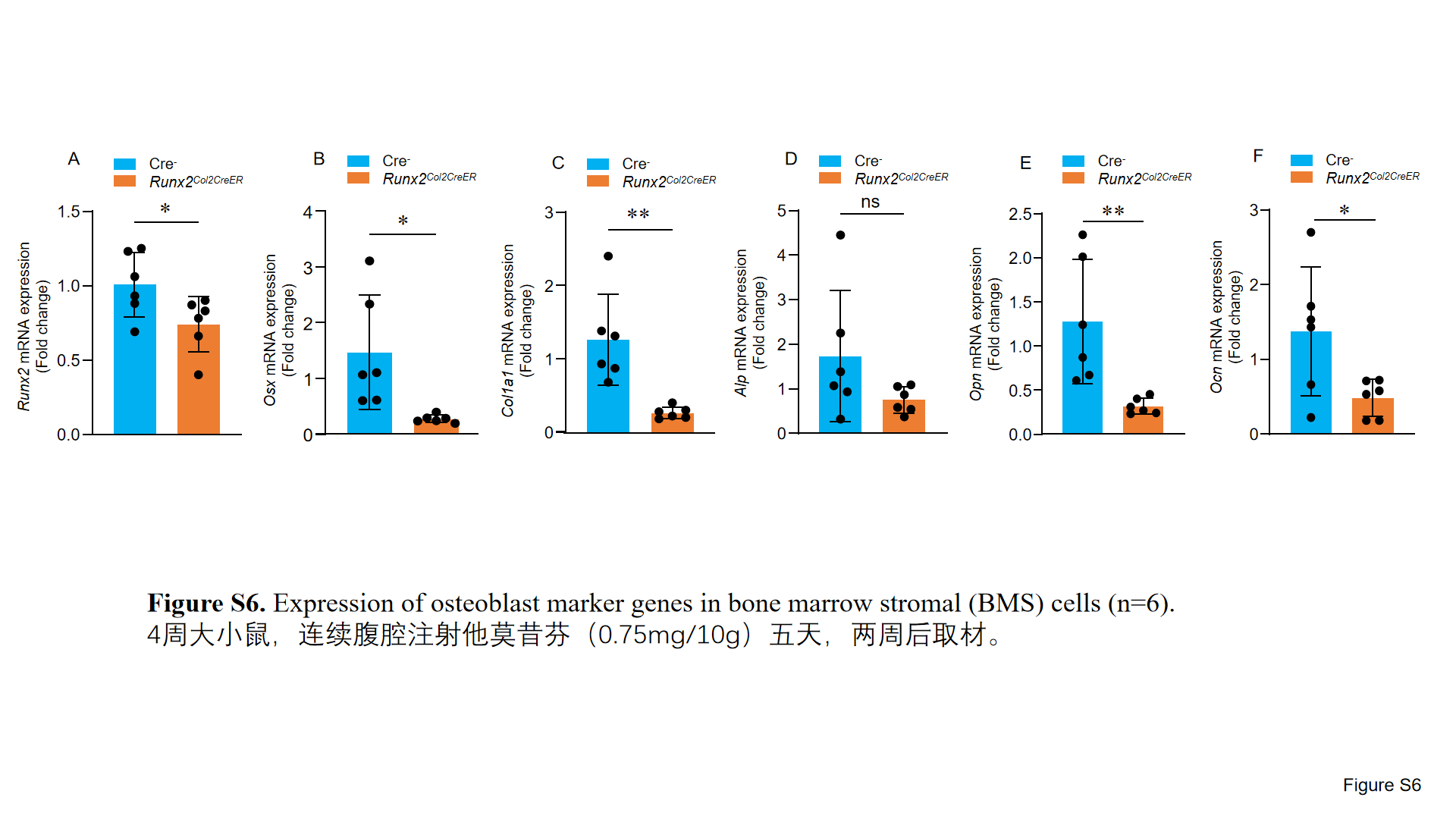


**Figure S5.** Expression of osteoblast marker genes in bone marrow stromal (BMS) cells. BMS cells were isolated from 6-week-old Cre^-^ and *Runx2^Col2CreER^* (*Runx2* cKO) mice. Expression of *Runx2*, *Osterix* (*Osx*), *Col1a1*, *Osteopontin* (*Opn*) and *Osteocalcin* (*Ocn*) was significantly reduced (A-C, E, F), while expression of *alkaline phosphatase* (*Alp*) was not significantly changed in BMS cells of *Runx2* cKO mice (D) (n=6).


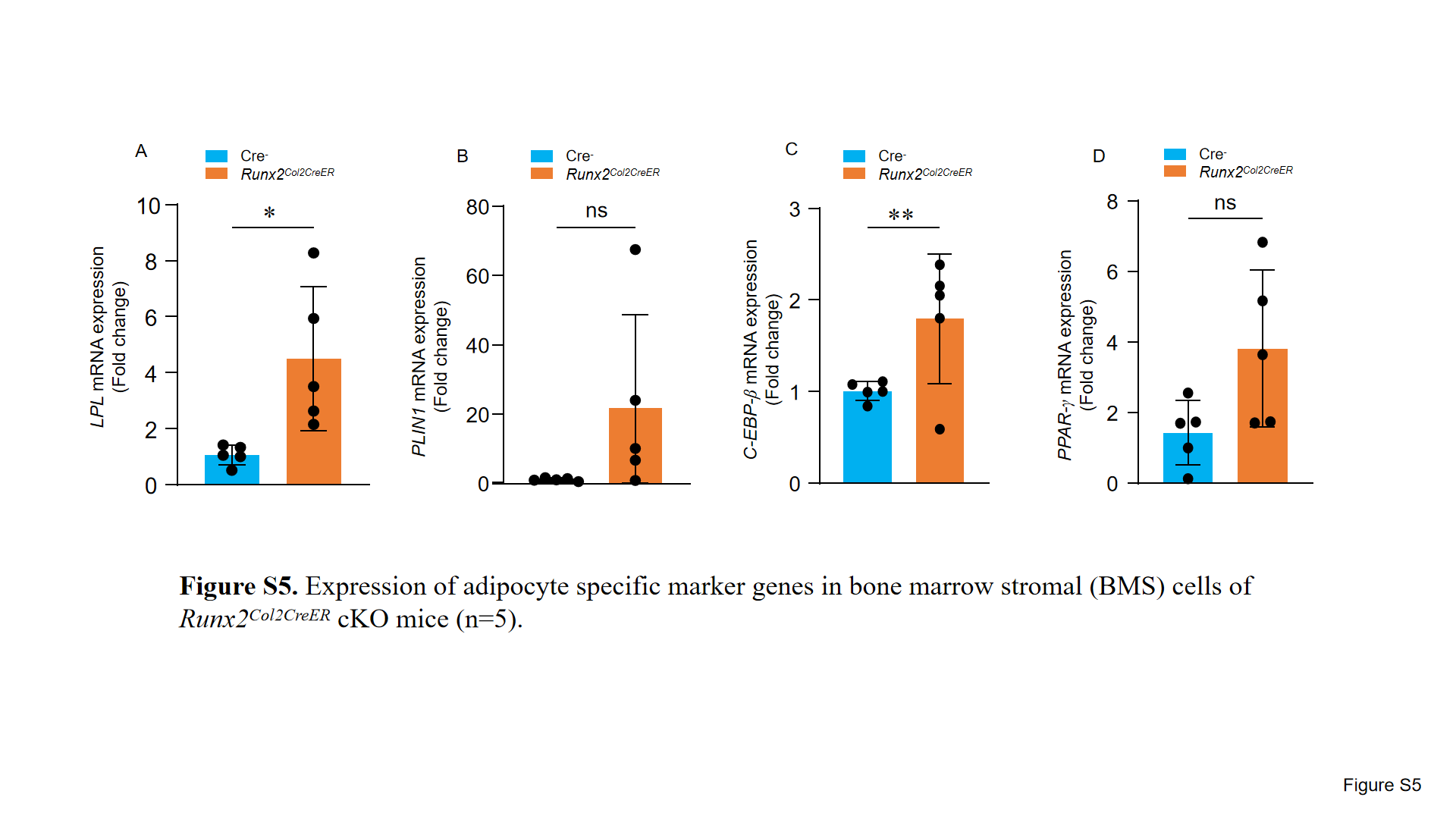


**Figure S6.** Expression of adipocyte marker genes in bone marrow stromal (BMS) cells of 4-week-old *Runx2^Col2CreER^* (*Runx2* cKO) mice. Expression of *Lipoprotein lipase* (*LPL*) and *CCAAT enhancer binding protein β* (*C-EBP-β*) in BMS cells was significantly upregulated (A, C), while expression of *Perilipin 1* (*PLIN1*) and *Peroxisome proliferator activated receptor γ* (*PPARγ*) was not significantly changed (B, D) in BMS cells of *Runx2* cKO mice (n=5).


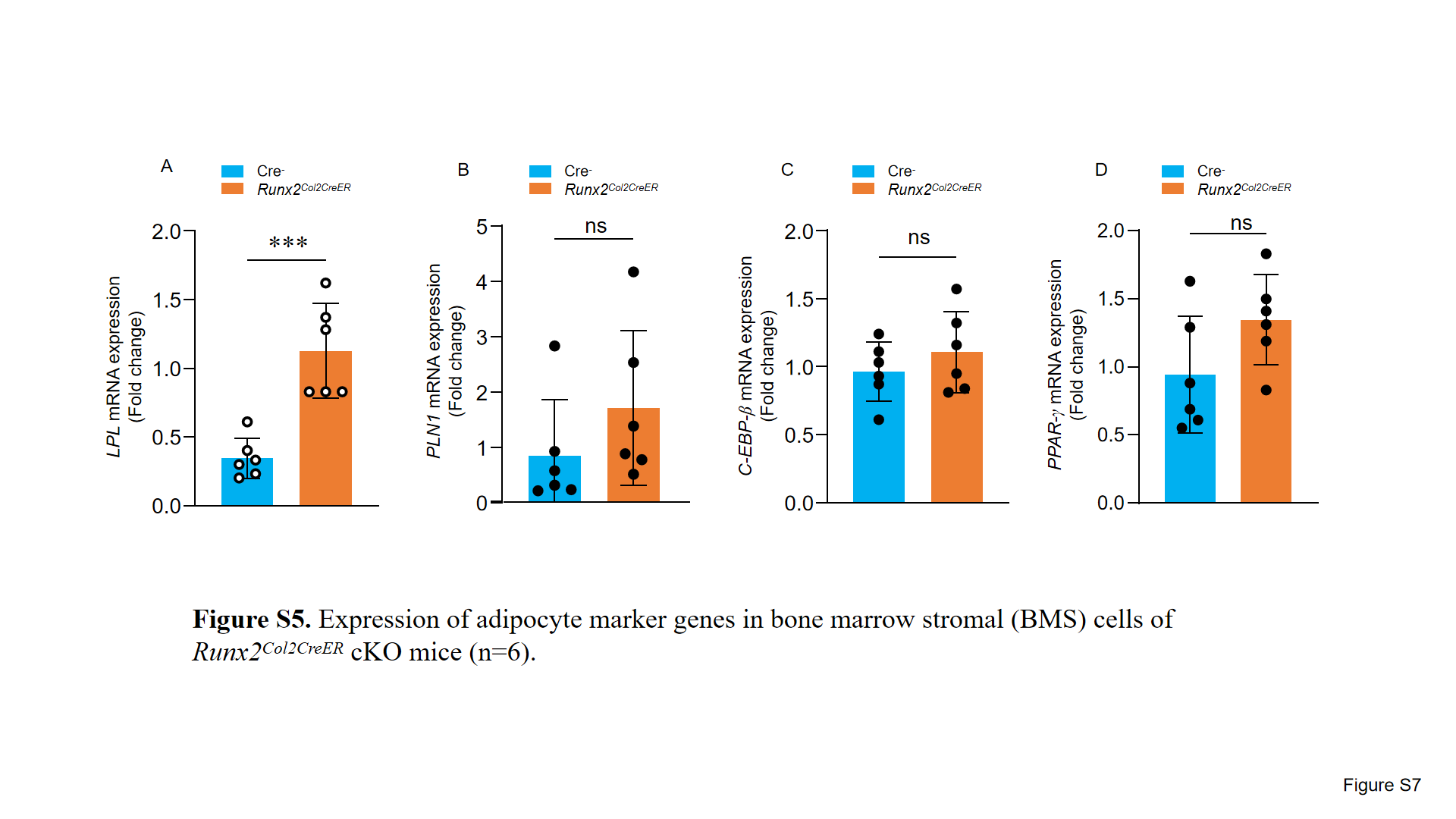


**Figure S7.** Expression of adipocyte marker genes in bone marrow stromal (BMS) cells of 6-week-old *Runx2^Col2CreER^* (*Runx2* cKO) mice. Expression of *Lipoprotein lipase* (*LPL*) was significantly upregulated (A), while expression of *Perilipin 1* (*PLIN1*), *CCAAT enhancer binding protein β* (*C-EBP-β*) and *Peroxisome proliferator activated receptor γ* (*PPARγ*) was not significantly changed (B-D) in BMS cells of *Runx2* cKO mice (n=6).

**
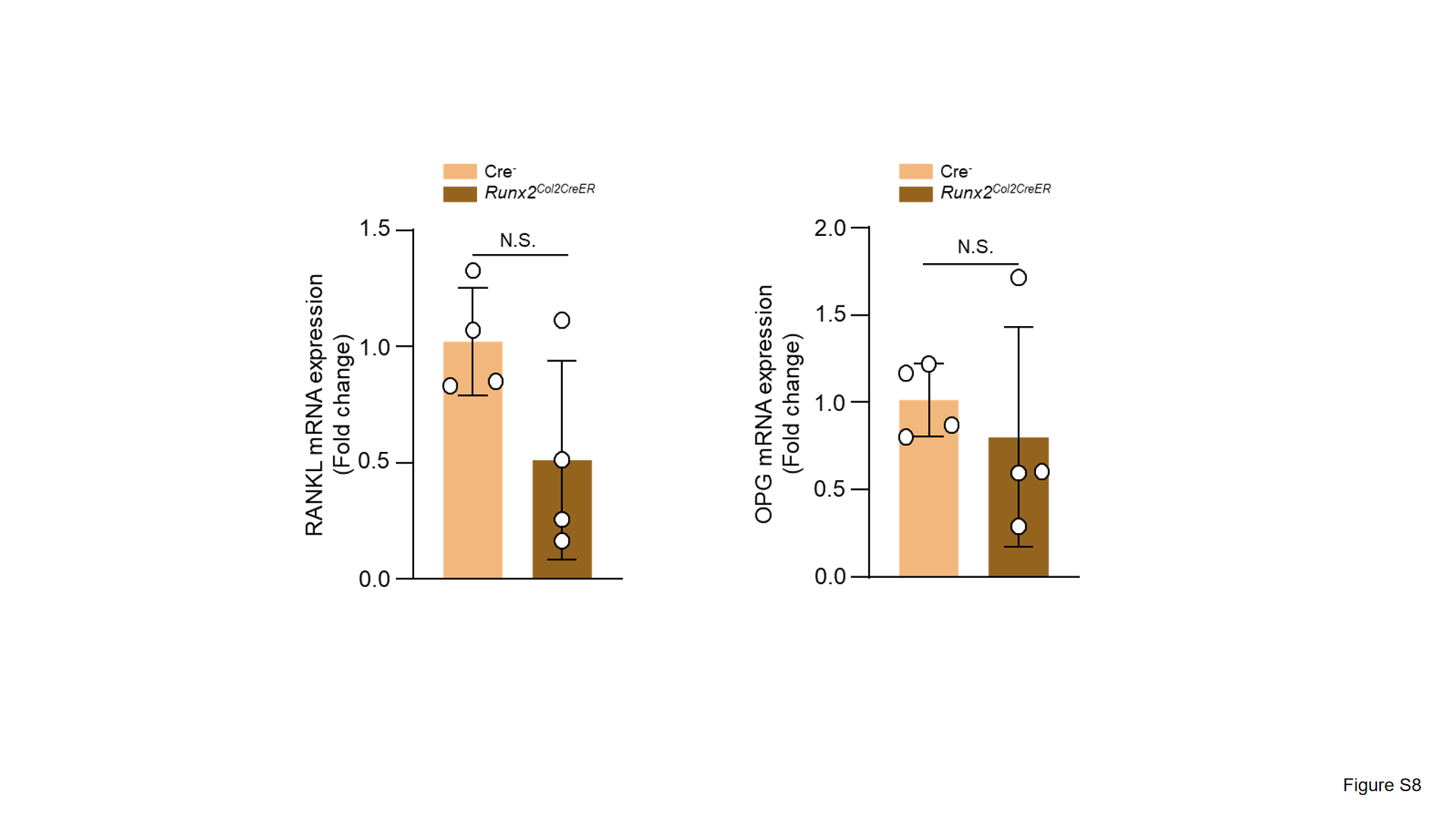
**

**Figure S8.** Expression of osteoclast marker genes in bone marrow stromal (BMS) cells of *Runx2^Col2CreER^* (*Runx2* cKO) mice. Expression of *RANKL* and *OPG* in BMS cells was examined by real-time PCR. No significant changes were detected.
